# Evaluation of familial mediterranean fever patients: a single center experience

**DOI:** 10.1186/1546-0096-13-S1-P111

**Published:** 2015-09-28

**Authors:** P Gulez, N Gulez, B Sozeri, F Hazan

**Affiliations:** 1 Dr. Behçet Uz Education and Research Hospital, Pediatric İmmunology, Izmir, Turkey

## Introduction

Familial Mediterranean Fever (FMF) is an autosomal recessive autoinflammatory disease due to mutations in MEFV, and characterized by recurrent acute attacks of fever and serosal inflamation. The disease mainly affects populations from the Mediterranean basin, especially Arabs, Turks, Jews, and Armenians. The diagnosis of the disease relies on clinical criteria, family history, and ethnic considerations, and genetic analysis of known mutations. Standart therapy for the prevention of acute attacks and also disease-related amiloidosis is colchicine. Valid therapeutic alternatives are anti-IL-1 agents in unresponder or noncompliant patients.

## Objectives

The aim of the study to evaluate familial mediterranean patients and to determine clinical characteristics, efficacy of colchicine drug and type of MEFV mutations.

Patients and methods: In this study we evaluated FMF patients diagnosed with the clinical criteria and genetic analysis. Their family history, consanguine, symptom-onset age, and age at the diagnosis, their complaints, the course of the disease, genetic analysis, therapy, complications, were recorded from their hospital records.

## Results

Total 355 FMF patients included the study. Their madian symptom-onset age was 6,0 year, and age at the diagnosis was 8,3 years. Almost a half of them had family history, their parents of 17,2% patients were relative. The most complaints were abdominal pain (72,1%), fever (70,4%), and artralgias (48,2%). 47,9% of patients had have M694V mutation, 69% of patients treated with colchicine, but 6 (2,5%) of them were resistance of colchicine, and anti-IL-1 agents used for therapy. In only one patient (0,3%) renal amiloidosis was developed. There was no complication observed due to therapies.

## Conclusion

We found the period from the disease onset to diagnosis is shorter, the responce to colchicine therapy is found more favorable than other studies. We also found unresponsiveness to colchicine therapy is smilarly with literature.

